# Open data from taxis and Bluetooth detectors to extract congestion and mobility patterns in Thessaloniki

**DOI:** 10.1016/j.dib.2023.108899

**Published:** 2023-01-13

**Authors:** Georgia Ayfantopoulou, Josep Maria Salanova Grau, Panagiotis Tzenos, Athanasios Tolikas

**Affiliations:** Centre for Research and Technology Hellas – CERTH, Hellenic Institute of Transport (HIT), 6th km Charilaou-Thermi Rd Thermi, Thessaloniki 57001, Greece

**Keywords:** Floating car data, Bluetooth detections data, Traffic status, Route travel time, Road link speed

## Abstract

Thessaloniki hosts one of the largest mobility living labs in Europe, aiming at fostering innovation to the mobility sector. Data is a key aspect of the living lab, allowing to depict mobility and congestion patterns to better manage traffic and support decision making. Most of the public and private stakeholders of the Thessaloniki mobility eco-system are part of the living lab and provide real-time data to the host of the living lab (CERTH-HIT), receiving added-value services from their participation. Thus, structured and unstructured Transportation and Mobility related datasets generated by various both conventional and innovative data sources, namely floating taxis and Bluetooth detectors, are being processed into “Thessaloniki's Smart Mobility Living Lab”, the data analysis and modelling laboratory of the Hellenic Institute of Transport (HIT). As most datasets are usually generated by high-rate and high-density machines, an intricate and efficient back-end pipeline is in place to ensure the proper collection, transformation, combination, and processing of such datasets in almost real time. In addition, many static datasets are kept and updated regularly.

**Specifications Table**

**Table:** 

Subject	Business, Management and decision sciences
Specific subject area	Transportation management using multi-source data to analyse the current status of network and provide services to the citizens and stakeholders.
Type of data	Real-time and historical datasets in JSON, XML, CSV, KML, MAP standards containing speed measurements and trip trajectories providing the sequence of locations or the origin and destination together with the relevant timestamps.
How the data were acquired	The data described were acquired: • via proprietary APIs that transmit data captured by the Bluetooth sensors installed in various traffic intersections throughout the city. The (anonymized) captured data are transferred via UPD protocol using the mobile network infrastructure (GPRS). • via access to APIs provided by the collaborative taxi association that provide the location data of the floating taxis, captured using the OBUs (Android tablets) capabilities. • via data processing by performing state of the art methodologies and algorithms on raw data (metadata).
Data format	Analyzed: JSON, XML, CSV, KML, MAP Filtered: JSON, XML, CSV, KML, MAP
Description of data collection	FCD: The taxi fleet consists of nearly 1000 vehicles moving continuously in the region of Thessaloniki. Each vehicle produces 1 GPS record per 10-12 s or 100 m of movement. Bluetooth: Each of the currently 43 Bluetooth sensors installed in the Region of Thessaloniki, captures the unique MAC address of a Bluetooth-enabled device when the latter enters its transmission range.
Data source location	• *Institution: Hellenic Institute of Transport (HIT), Centre for Research and Technology Hellas - CERTH* • *City/Town/Region: Thessaloniki* • *Country: Greece*
Data accessibility	Repository name: CERTH-HIT OpenData Hub Greece Data identification number: https://doi.org/10.25504/FAIRsharing.551691 Direct URL to data: https://opendata.imet.gr/dataset Instructions for accessing these data: The data can either be downloaded locally or via an API (with paging).

**Value of the Data**

• Floating car data from taxis containing speed and map-matched location and Bluetooth detection data are used to extract congestion patterns. These are used to support traffic management in the city by being an umbrella above the three technology providers and allowing the city to coordinate their efforts.
• Floating car data containing location and vehicle status (with customer or not) and Bluetooth detection data are used to generate mobility patterns, which are used to feed transport modelling and support the city in decision making related to mobility (e.g. these were used to define the network of micro-mobility stations in the city, including location and size but also expected rebalancing needs).
• All these datasets are used by OR and AI algorithms to optimize mobility systems (e.g. location of stations, areas for on-demand services)

## 1 Data Description

The datasets contained in the repository are described below. In Table 1 the datasets described and the relevant data fields are summarised.

**Table 1:** Descriptions of the datasets and data fields available.

		Data fields			

Dataset name	Description	Field name	Field description	Available formats	Historical dataset
fcd-gps	Floating car data collected from the biggest taxi fleet in Thessaloniki	recorded_timestamp	The time and date the FCD entry was recorded (local time)	JSON, XML, CSV, KML and MAP	fcd-gps-historical (in TXT)
		lat	Location: Latitude		
		lon	Location: Longitude		
		altitude	The altitude of the vehicle		
		speed	The GPS generated speed (km/h)		
		orientation	Orientation of the vehicle (degrees)		

itravel-detections	Aggregated number of the total detections per “iTravel” Bluetooth sensors	device_id	The identification of the Bluetooth sensor (corresponds to the id of the dataset itravel-devices)	JSON, XML and CSV	itravel-detections-historical (in TXT)
		NumberOfRecords	The aggregated number of detections per device		
		timestamp	The time and date of the detections (local time)		

network-speed	Calculated average speed on the road network in Thessaloniki	Link_id	The OpenStreetMap road segment “wayId”	JSON, XML and CSV	network-speed-historical (in TXT)
		Link_Direction	The travel direction of the road segment		
		Timestamp	The time and date (local time)		
		Speed	The calculated average speed		
		UniqueEntries	The total number of FCD used for the calculation		

network-congestion	Congestion in the road network of the Thessaloniki	Link_id	The OpenStreetMap road segment “wayId”	JSON, XML and CSV	-
		Link_Direction	The travel direction of the road segment		
		Timestamp	The time and date (local time)		
		Congestion	The level of congestion – low, medium, high		

itravel-traveltimes	The travel time across predefined paths in Thessaloniki using the Bluetooth detections	Path_id	The id of the path (corresponds to the id in the itravel-paths dataset)	JSON, XML and CSV	itravel-traveltimes-historical (in TXT)
		Timestamp	The timeframe of the calculation		
		Duration	The travel time duration (seconds)		

fcd-traveltimes	The travel time across predefined paths in Thessaloniki using FCD	Path_id	The id of the path (corresponds to the id in the itravel-paths dataset)	JSON, XML and CSV	fcd-traveltimes-historical (in TXT)
		Timestamp	The timeframe of the calculation		
		Duration	The travel time duration (seconds)		
datafusion-traveltimes	The travel time across predefined paths in Thessaloniki using both the Bluetooth detections and FCD	Path_id	The id of the path (corresponds to the id in the itravel-paths dataset)	JSON, XML and CSV	datafusion-traveltimes-historical (in TXT)
		Timestamp	The timeframe of the calculation		
		Duration	The travel time duration (seconds)		

itravel-devices	The static data of the Bluetooth devices’ characteristics	device_id	The unique identification of the device	JSON, XML, CSV, KML and MAP	-
		device_Name	The name of the device		
		lat	The latitude of the installation location		
		lon	The longitude of the installation location		

itravel-paths	The geolocation of the predefined paths in Thessaloniki between the iTravel Bluetooth devices	Path_id	The unique identification of the path	JSON, XML, CSV, KML and MAP	-
		Path_Name	The path's descriptive name		
		Path_origin_device_id	The origin's device id		
		Path_destination_ device_id	The destination's device id		
		polyline	The path in polyline format		

### 1.1 fcd-gps

This dataset contains anonymized floating car data collected from a taxi fleet of over one thousand vehicles that operate in the city of Thessaloniki. The information available from this dataset is the location of the vehicle (longitude, longitude), the speed, the altitude, the orientation, and the timestamp the data was recorded. This dataset is available in JSON, XML, CSV, KML and MAP format and is updated in almost real time. A historical dataset, updated every month, is also available in TXT (tab-delimited text file).

### 1.2 itravel-detections

In this dataset, the aggregated number of detections per iTravel Bluetooth device is stored. The fields included are device id (that corresponds to the id of the dataset itravel-devices described below), number of records and timestamp. A historical dataset (itravel-detections-historical) in TXT format is also available and updated monthly.

### 1.3 network-speed

This dataset contains the current speeds of the road network in Thessaloniki as calculated using the fcd-gps dataset. OpenStreetMap (OSM) road segments are used for the identification of the segments. The dataset contains the Link_id (OSM road segment), Link_direction (the direction of the road segment), timestamp, speed (calculated) and uniqueEntries (number of FCD used for the calculation). This dataset is available as JSON, XML or CSV and is updated every 15 min. Lastly, a historical dataset (network-speed-historical) is also available in TXT (tab-delimited text file) format and updated monthly.

### 1.4 network-congestion

Similarly, to the network speeds, this dataset is also calculated using the fcd-gps dataset. As the name suggests, this dataset depicts the congestion in the road network of the Thessaloniki. It contains the fields: Link_id (OSM road segment), Link_Direction (direction of the road segment), Timestamp, Congestion (the level of congestion – low, medium, high). It is available in JSON, XML or CSV format and is updated every 15 min.

### 1.5 itravel-traveltimes

This dataset is produced using the Bluetooth detections (itravel-detections dataset). It is updated in almost real time and contains the path id (corresponds to the id in the itravel-paths dataset below), the timestamp and the duration. Historical dataset (itravel-traveltimes-historical) is also available in TXT format and updated monthly.

### 1.6 fcd-traveltimes

This dataset is similar to the travel-traveltimes dataset described above. In this case the FCD data are used to calculate the travel time for the predefined paths. It is available in JSON, XML and CSV format alongside the historical dataset (fcd-traveltimes-historical).

### 1.7 datafusion-traveltimes

This dataset is produced using both the iTravel Bluetooth detections and FCD data to calculate the current travel times for the selected paths. It is available in JSON, XML and CSV format. A historical dataset (datafusion-traveltimes-historical) is available in TXT format and updated monthly.

### 1.8 itravel-devices

This dataset holds the static data of the Bluetooth detection devices’ characteristics. Namely, the fields contained in the dataset are the device id, the device name, and the location (longitude, latitude).

### 1.9 itravel-paths

This is a static dataset of the geolocation of the predefined paths in Thessaloniki between the iTravel Bluetooth devices. It contains the path id, the path name, the ids of origin and destination Bluetooth id and the coordinates of the path using a polyline. It is available in XML, JSON, CSV, KML and MAP formats.

## 2 Experimental Design, Materials and Methods

The data described in the previous section were collected through various sources, namely detectors placed in strategic locations and floating cars (specifically taxis) that traverse the city of Thessaloniki. The methodology of assessing and cataloguing these datasets is described in [1,2]. Furthermore, relevant urban mobility indicators can be generated as presented by Mistakis et al in [3]. All the open datasets provided in the repository are anonymized and strictly follow the GDPR guidelines.

### 2.1 Floating Car Data

This dataset (**fcd-gps**) contains anonymized floating car data collected from the biggest taxi fleet association (Taxiway) consisting of over one thousand vehicles that operate in the city of Thessaloniki. Every moving vehicle produces and transmits 1 GPS record per 10-12 s or 100 m of movement. In average nearly 2 thousand new FCD records per minute are being generated while the dataset is updated in almost real-time.

### 2.2 Bluetooth Detections

Over 43 Bluetooth detection devices are placed throughout the major road junctions of the city of Thessaloniki. The **itravel-detections** dataset holds the aggregated detections of each Bluetooth device at a specific timeframe. This data is derived from detection records of MAC addresses of devices that are in range of the detector and are in discoverable mode. The captured data are subsequently anonymized and transmitted to the server via the UPD protocol using the mobile network infrastructure (GPRS).

### 2.3 Network Status

Utilizing the FCD and Bluetooth detections datasets through a series of calculation and state-of-the-art methodology, valuable information on the status of the network can be produced, namely the average speeds and congestion. The **network-speed** dataset provides accurate and real-time traffic information in Thessaloniki, Greece by estimating the average moving speed of the vehicles on the road network. The speed estimations are produced every 15 min although it is possible for this frequency value to change in the future. Initially, all the FCD records are processed appropriately to remove any data that would allow unauthorized or voluntary user identification and are filtered to remove any erroneous entries with extraordinary speeds or unaligned coordinates as well as entries generated by faulty GPS receivers. A specific algorithm then, that considers the network topology (monographs, types of roads, etc.), each record (point) is mapped to the part of the road network to which it is most likely to belong with the highest degree of certainty [4]. Lastly, for each section (segment) of the road network, proper statistical analysis is performed to provide a safe estimation of the average speed at which vehicle traffic is conducted on it. Similarly, the **network-congestion** dataset provides traffic information in a qualitative (low, medium, high) manner based on the speed calculated for the network-speed dataset described above. Specifically, the calculated speed is divided with the free flow speed of the road segment and depending on a threshold the qualitative value is produced. It is important to notice that OpenStreetMap (OSM) road segments are used for the identification of the segments where the Link_id contained in these datasets correspond to the wayId used in the OSM architecture.

### 2.4 Travel Times

Similarly, to the network status datasets, the travel times datasets are calculated by feeding the FCD and Bluetooth detections (as well as a combination of both) through in-house developed algorithms [5] that output the duration in seconds for predefined paths in Thessaloniki. These paths are stored in the static dataset **itravel-paths.** The origins and destinations of these paths are defined by the locations of the Bluetooth sensors that are stored in the static **itravel-devices** dataset. The datasets **itravel-traveltimes** and **fdc-traveltimes** use as input the FCD and Bluetooth detections respectively in order to calculate the travel time. Furthermore, the outputs of both methodologies are combined in an effort to increase the overall system's output accuracy and credibility (**datafusion-traveltimes)**. All three datasets are updated every 15 min.

## Ethics Statements

All described data in this paper strictly follow the GDPR guidelines for personal data. No publicly available data can be traced back to an individual. This submission follows the ethical requirement for publication in Data in Brief.

## CRediT authorship contribution statement

**Georgia Ayfantopoulou:** Conceptualization, Supervision, Project administration. **Josep Maria Salanova Grau:** Conceptualization, Supervision, Methodology, Validation, Investigation, Writing – review & editing. **Panagiotis Tzenos:** Conceptualization, Methodology, Software, Data curation, Visualization, Formal analysis. **Athanasios Tolikas:** Writing – original draft, Software, Visualization.

## Declaration of Competing Interest

The authors declare that they have no known competing financial interests or personal relationships that could have appeared to influence the work reported in this paper.

## Data Availability

fcd-gps (Original data) (CERTH-HIT OpenData Hub Greece). datafusion-traveltimes (Original data) (CERTH-HIT OpenData Hub Greece). network-speed-historical (Original data) (CERTH-HIT OpenData Hub Greece). itravel-traveltimes-historical (Original data) (CERTH-HIT OpenData Hub Greece). itravel-detections-historical (Original data) (CERTH-HIT OpenData Hub Greece). fcd-traveltimes-historical (Original data) (CERTH-HIT OpenData Hub Greece). fcd-gps-historical (Original data) (CERTH-HIT OpenData Hub Greece). datafusion-traveltimes-historical (Original data) (CERTH-HIT OpenData Hub Greece). network-speed (Original data) (CERTH-HIT OpenData Hub Greece). network-congestion (Original data) (CERTH-HIT OpenData Hub Greece). itravel-traveltimes (Original data) (CERTH-HIT OpenData Hub Greece). itravel-paths (Original data) (CERTH-HIT OpenData Hub Greece). itravel-devices (Original data) (CERTH-HIT OpenData Hub Greece). itravel-detections (Original data) (CERTH-HIT OpenData Hub Greece). fcd-traveltimes (Original data) (CERTH-HIT OpenData Hub Greece).
